# MicroRNA-320a promotes 5-FU resistance in human pancreatic cancer cells

**DOI:** 10.1038/srep27641

**Published:** 2016-06-09

**Authors:** Weibin Wang, Lijun Zhao, Xueju Wei, Lanlan Wang, Siqi Liu, Yu Yang, Fang Wang, Guotao Sun, Junwu Zhang, Yanni Ma, Yupei Zhao, Jia Yu

**Affiliations:** 1 Department of General Surgery, Peking Union Medical College Hospital, Chinese Academy of Medical Science & Peking Union Medical College, Beijing 100730, PR China; 2 Department of Biochemistry, Institute of Basic Medical Sciences, Chinese Academy of Medical Sciences, School of Basic Medicine Peking Union Medical College, Beijing 100005, PR China; 3 Institute of Molecular Medicine, Medical School, Henan University, KaiFeng, 475000, PR China

## Abstract

The drug-resistance of pancreatic cancer cells results in poor therapeutic effect. To predict the therapeutic effect of the chemotherapy drugs to specific patients and to reverse the resistance of pancreatic cancer cells are critical for chemotherapy of pancreatic cancer. MicroRNAs (miRNAs) have been reported to play important roles in the genesis of drug-resistance of various cancer types. There are also many advantages of miRNAs in diagnosis and therapy of disease. Although several miRNAs regulating 5-Fluorouracil (5-FU) resistance in human pancreatic cancer have been reported, the detailed molecular mechanism remains to be determined. In this study, we found that miR-320a was significantly up-regulated in 5-FU resistant pancreatic cancer cells. Over-expression of miR-320a strongly contributed to pathogenesis of pancreatic cancer, which was represented by the increased proliferation, invasion, metastasis, drug-resistance characteristics and the epithelial-to-mesenchymal transition. Furthermore, we demonstrated that miR-320a was able to bind to 3′UTR of PDCD4 mRNA, and mediated its down-regulation in 5-FU resistance of human pancreatic cancer cells. Whereas restoration of PDCD4 expression could partially attenuate the function of miR-320a in pancreatic cancer. Taken together, our study demonstrated that miR-320a played important role in regulating 5-FU resistance by targeting PDCD4 and might be developed as new therapeutic target for pancreatic cancer.

The world has seen a booming of pancreatic cancer during the past years. The latest surveys suggest that the incidence of pancreatic cancer increased three times during the past 10 years^1^, ranking the fourth disease of cancer mortality all over the world, which also ranks the seventh in China^2^,^3^. The majority of patients diagnosed with pancreatic cancer are in the terminal period, and the rate of postoperative local recurrence was above 85% after surgery. Therefore chemotherapy becomes one of the important treatment for patients with pancreatic cancer. 5-FU is the earliest chemotherapy drug for treating pancreatic cancer, but the drug resistance against 5-FU are clinical widespread, which is one of the important factors affecting the effect of chemotherapy. Therefore, understanding the molecular mechanisms of 5-FU resistance that are associated with its aggressiveness and high propensity for metastasis and developing novel therapeutic targets for pancreatic cancer are imperative.

Over the years, resistance of chemotherapy drugs used to cure pancreatic cancer has been intensively studied and some underlying mechanisms have been clarified. The suppression of chemotherapy drug transport and metabolism in the tumor cells and anti-apoptotic effect of tumor cells are considered to be the common reasons for the drug resistance of tumor cells^4^. In addition, studies have shown that epithelial-mesenchymal transition (EMT), cancer stem cells and microRNAs (miRNAs) play key roles in the formation of drug resistance during the pancreatic cancer chemotherapy^5^,^6^,^7^. For most epithelial tumors, progression toward malignancy is accompanied by a loss of epithelial differentiation and a shift towards mesenchymal phenotype^8^. During the acquisition of EMT characteristics, cancer cells lose the expression of proteins that promote cell-cell contact such as E-cadherin and β-catenin, and gain the expression of mesenchymal markers such as Vimentin, Fibronectin, and N-cadherin, leading to enhanced cancer cell migration and invasion. EMT has been shown to be important on conferring drug resistance characteristics to cancer cells against conventional therapeutics including taxol, vincristine, oxaliplatin, or epidermal growth factor receptor (EGFR) targeted therapy^9^.

Moreover, emerging evidence implicates the critical role of miRNAs because they are key regulatory molecules in various biological and pathological processes including EMT. These small, noncoding molecules elicit their regulatory effects by imperfectly binding to the 3′untranslated region (3′UTR) of target mRNA, causing either degradation of mRNA or inhibition of their translation to functional proteins^10^,^11^. Many studies have established this concept by discovering the up-regulation or down-regulation of specific miRNAs in various types of cancer and identifying some of their molecular targets^12^,^13^,^14^. In recent years, miRNAs have been identified to enhance several aspects of pancreatic cancer pathogenesis, including proliferation, invasion, metastasis and drug resistance characteristics^15^.

Emerging evidence suggests that the expression of several miRNA genes is fundamental to the acquisition of the EMT phenotype and aggressiveness of tumor cell is also regulated by miRNAs^16^,^17^. What’s more, the process of chemotherapy drugs resistance in tumor cells is associated with the changes of specific miRNAs expression, which control the resistance of tumor cells through regulating the specific mRNA molecules. The expression of some drug resistance related miRNAs is also closely correlative with the survival of patients with pancreatic cancer chemotherapy.

In our studies, we find some miRNAs are up-regulated in 5-FU drug-resistant PATU8988 pancreatic cancer cells, such as miR-320a, miR-1291, miR-615-5p and so on. Although the role of miRNAs in cancer has been documented, there are very few studies documenting the cellular consequence due to target inactivation or re-expression of specific miRNA in pancreatic cancer cells. MiR-320a has been reported to be a potential biomarkers for colorectal carcinoma, and acts as a prognostic factor and inhibits metastasis of salivary adenoid cystic carcinoma by targeting ITGB3^18^,^19^. In this study, we identify that miR-320a plays an important role in promoting 5-FU resistance of human pancreatic cancer cells by targeting PDCD4, and facilitating several aspects of pancreatic cancer pathogenesis, including proliferation, invasion, metastasis, drug-resistance characteristics and the epithelial-to-mesenchymal transition. Furthermore, the expression of miRNAs and the specific targets can predict the effectiveness of the chemotherapy drugs for particular patients, which also implies miR-320a can be developed as a new prognostic marker for chemotherapy of pancreatic cancer.

## Results

### Establishment of 5-FU-resistant cell lines and identification of differentially expressed miRNAs

**Figure 1:**
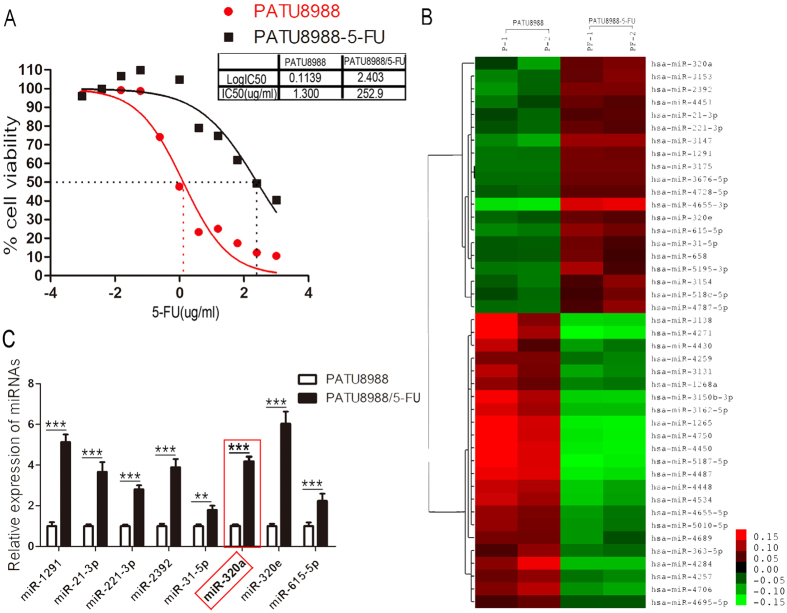
Up-regulation of miR-320a in PATU8988/5-FU cells. (**A**) Representative curves of growth-inhibitory effects in 5-FU resistant PATU8988/5-FU and PATU8988 cells. IC50, inhibitory concentration 50. (**B**) Microarray of miRNA expression profile of PATU8988/5-FU and PATU8988 cells. Each sample is biologically duplicated. (**C**) Up-regulated microRNAs between 5-FU resistant PATU8988/5-FU cells and its parental PATU8988 cells. All data are presented as the means ± SD. Student’s *t* test (two-tailed) was performed to analyze data from the experiments in triplicate. *P* values < 0.05 were considered significant, as indicated by the asterisks (**p < 0.01, ***p < 0.001).

To systematically screen the miRNAs involved in the induction of 5-FU drug resistance, we firstly created 5-FU-resistant pancreatic cancer cell lines and analyzed the expression profile of miRNAs using miRNA microarray. To establish 5-FU-resistant pancreatic cancer cell lines, PATU8988 cells were treated with 5-FU for one month and the resistant cells (PATU8988/5-FU) were selected. We also examined its response to 5-FU using MTT assay. And the IC50 values were 1.300 μg/ml for PATU8988 cells and 252.9 μg/ml for PATU8988/5-FU ones. Thus the resistance of PATU8988/5-FU cells to 5-FU was estimated to be approximately two hundred times higher than that of the parental cells (Fig. 1A). Further, we investigated the expression profile of miRNAs in PATU8988/5-FU and PATU8988 cells through microarray to find the differentially expressed miRNAs. The results showed that there were 20 miRNAs including miR-320a, miR-3153, miR-21, miR-221, miR-320e, significantly up-regulated in PATU8988/5-FU cells compared with PATU8988 cells. Meanwhile, there were also 23 miRNAs including miR-3138, miR-363, miR-4271, significantly down-regulated in PATU8988/5-FU cells (Fig. 1B). The results from miRNA microarray were validated by RT-PCR analysis for representative miRNA with varying expression profiles. The RT-PCR results of several miRNAs up-regulated in 5-FU-resistant cells were shown in Fig. 1C (*p < 0.05, **p < 0.01, ***p < 0.0001). While miR-320a was significantly up-regulated in the PATU8988/5-FU cells compared with that in PATU8988 cells with a fold change of 5.2 (***p < 0.0001). These results led us to speculate that up-regulation of miR-320a may be associated with 5-FU resistance of pancreatic cancer cells. The resistant cells were continuously maintained in culture medium containing 5-FU for the following study.

### Over-expression of miR-320a induces resistance to 5-FU in pancreatic cancer cells

**Figure 2:**
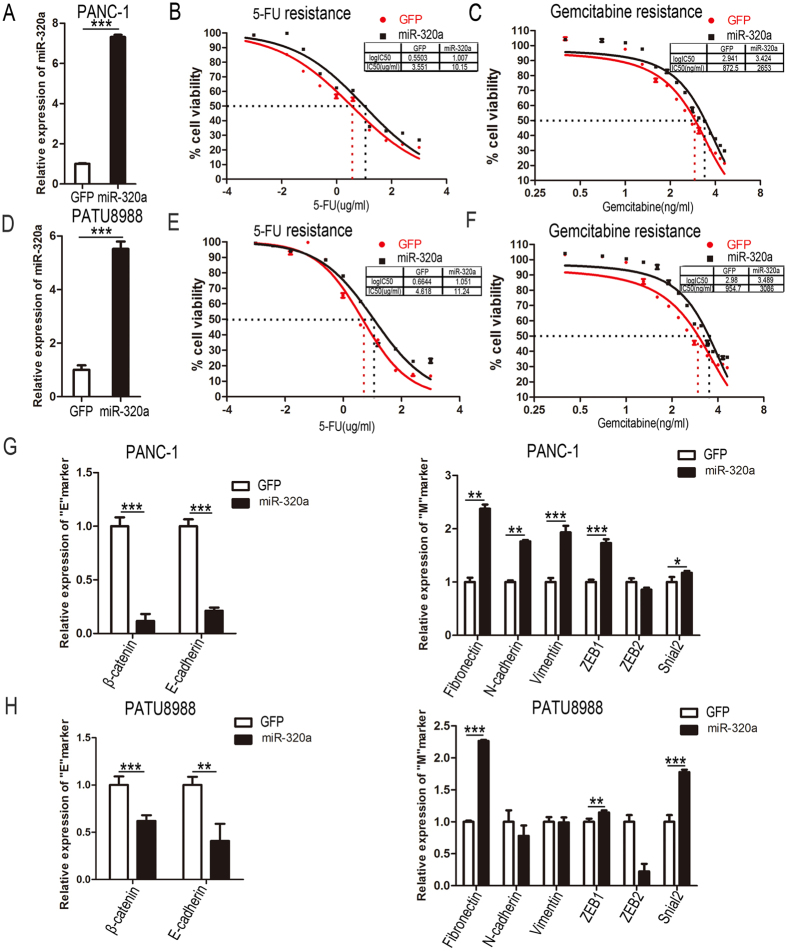
Over-expression of miR-320a induces resistance to 5-FU in pancreatic cancer cells. (**A**) RT-PCR results showed the expression of miR-320a in PANC-1 cells after lentivirus infection with lenti_GFP and lenti_miR-320a. (**B**) Representative curves of growth-inhibitory effects of 5-FU in PANC-1 cells which infected with lenti_miR-320a and lenti_GFP. (**C**) Representative curves of growth-inhibitory effects of gemcitabine in PANC-1 cells which infected with lenti_miR-320a and lenti_GFP. (**D**) RT-PCR results of the expression of miR-320a in PATU8988 cells after lentivirus infection with lenti_GFP and lenti_miR-320a. (**E**) Representative curves of growth-inhibitory effects of 5-FU in PATU8988 cells after infecting with lenti_miR-320a and lenti_GFP. (**F**) Representative curves of growth-inhibitory effects of gemcitabine in PATU8988cells which infected with lenti_miR-320a and lenti_GFP. (**G,H**) Real-time PCR assay was conducted to detect the expression of “EMT” markers in PANC-1 cells and PATU8988 cells. Left panel: the expression of β-cadherin, E-cadherin, Right panel: the expression of Fibronectin N-cadherin, Vimentin, ZEB1, ZEB2 and Snail in PANC-1 cells. All data are presented as the means ± SD. Student’s *t* test (two-tailed) was performed to analyze data from the experiments in triplicate. *P* values < 0.05 were considered significant, as indicated by the asterisks (*p < 0.05, **p < 0.01, ***p < 0.001).

To determine the contribution of miR-320a to the 5-FU resistance in pancreatic cancer cells, PATU8988 and PANC-1 cells were transduced with lenti_miR-320a and lenti_GFP (as a control) respectively, and treated with 5-FU. MiR-320a was overexpressed successfully in PATU8988 and PANC-1 cells, determined by quantitative RT-PCR (Fig. 2A,D). As a result, miR-320a decreased the sensitivity to 5-FU in both PANC-1 and PATU8988 cells compared with the control. IC50 was evaluated as 10.15 μg/ml for miR-320a over-expression group and 3.551 μg/ml for the control in PANC-1 cells, and 11.24 μg/ml vs 4.618 μg/ml in PATU8988 cells (Fig. 2B,E). Thus the IC50 values in miR-320a overexpressed cells were approximately 2.6 times higher than that of the control cells. Gemcitabine was also one of the most popular chemotherapy drugs used in pancreatic cancer treatment, so we also detected the effect of miR-320a in regulating gemcitabine resistance. We found that IC50 of gemcitabine was evaluated as 2653 ng/ml for miR-320a over-expression group and 872.5 ng/ml for the control in PANC-1 cells, and 3066 ng/ml vs 954.7 ng/ml in PATU8988 cells (Fig. 2C,F). Thus the IC50 values in miR-320a overexpressed cells were both higher than that of the control cells. According to these data, expansion of miR-320a might have an important role for the acquisition of 5-FU and gemcitabine resistance.

### Over-expression of miR-320a promotes EMT molecular marker changes in pancreatic cancer cells

Emerging evidence also suggest that there is a molecular link between EMT phenotype with chemo-or radio-resistance^7^,^20^,^21^,^22^,^23^. Thus, an understanding of the molecular biology of EMT in pancreatic cancer may provide insights into the mechanisms of tumor proliferation, invasion and metastatic progression and facilitate the development of alternative therapeutic approaches to improve the treatment outcomes for patients suffering from pancreatic cancer^24^. To investigate whether miR-320a promote EMT to facilitate drug-resistance of pancreatic cancer cells, we measured the mRNA levels of EMT markers using RT-PCR in PANC-1 and PATU8988 cells. We found that epithelial molecules, such as β-cadherin and E-cadherin mRNA, were down-regulated, while the mRNA levels of mesenchymal markers including Fibronectin, N-cadherin, Vimentin, ZEB1 and Snail2 were up-regulated in PANC-1 cells with enforced expression of miR-320a (Fig. 2G). And the similar results were found in PATU8988 cells (Fig. 2H). These results further suggested that miR-320a promoted pancreatic cancer cells to acquire a mesenchymal phenotype to facilitate drug resistance genesis.

### Over-expression of miR-320a promotes proliferation and migration of pancreatic cancer cells

**Figure 3:**
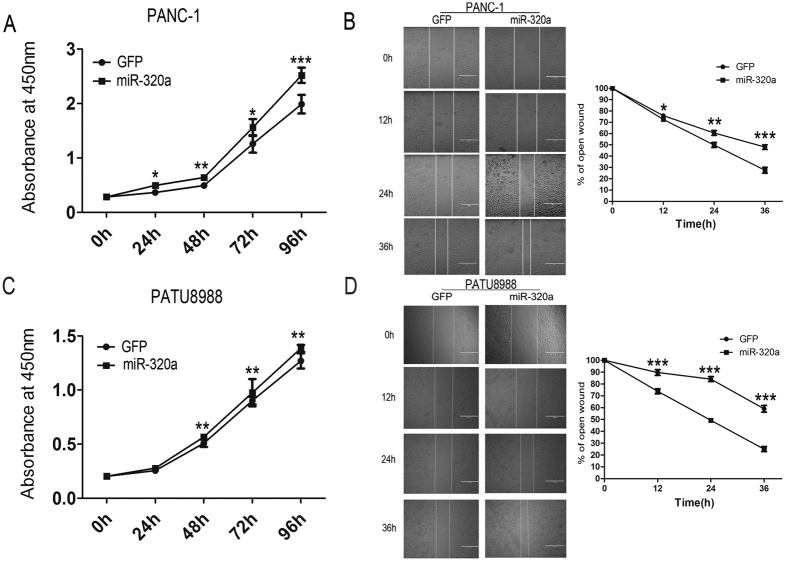
Over-expression of miR-320a promotes pancreatic cancer cell proliferation and migration. (**A**) Proliferation assays by cck-8 at 0, 24, 48, 72 and 96 h, after infecting with lenti_miR-320a or lenti_GFP in PANC-1 cells. (**B**) Left panel: PANC-1 cells were infected with lenti_miR-320a or lenti_GFP for 36 h, and wounds were made. Bar, 100 uM. Right panel: The relative ratio of wound closure per field is shown at 0, 12, 24 and 36 h. (**C**) Proliferation assays by cck-8 at 0, 24, 48, 72 and 96 h, after infecting with lenti_miR-320a or lenti_GFP in PATU8988 cells. (**D**) Left panel: PATU8988 cells were infected with lenti_miR-320a or lenti_GFP for 36 h, and wounds were made. Bar, 100 uM. Right panel: The relative ratio of wound opening per field is shown at 0, 12, 24 and 36 h. All data are presented as the means ± SD. Student’s *t* test (two-tailed) was performed to analyze data from the experiments in triplicate. *P* values < 0.05 were considered significant, as indicated by the asterisks (*p < 0.05, **p < 0.01, ***p < 0.001).

Recent studies have shown that EMT is associated with drug resistance and cancer cell metastasis^9^,^25^. To explore whether miR-320a regulates the aggressive characteristics of pancreatic cancer cells, we measured the proliferation and migration in PANC-1 and PATU8988 cells with miR-320a over-expression. The proliferation rate of pancreatic cells was measured at 0 h, 24 h, 48 h, 72 h and 96 h respectively, and the results showed that miR-320a promoted the growth of both two pancreatic cell lines PANC-1 and PATU8988(Fig. 3A,C). To further validate whether pancreatic cancer cells with miR-320a over-expression had enhanced motility, we detected cell migration using wound-healing assay. Our migration results showed that over-expression of miR-320a increased cell migration compared with their control cells in PANC-1 cells and PATU8988 cells. We found that miR-320a significantly promoted the migration rate during the opening of a wound created in a confluent monolayer (Fig. 3B) in PANC-1 and PATU8988 cells (Fig. 3D). In line with these findings, we demonstrated that miR-320a promoted proliferation and migration of pancreatic cancer cells.

### Over-expression of miR-320a promotes invasion of pancreatic cancer cells

**Figure 4:**
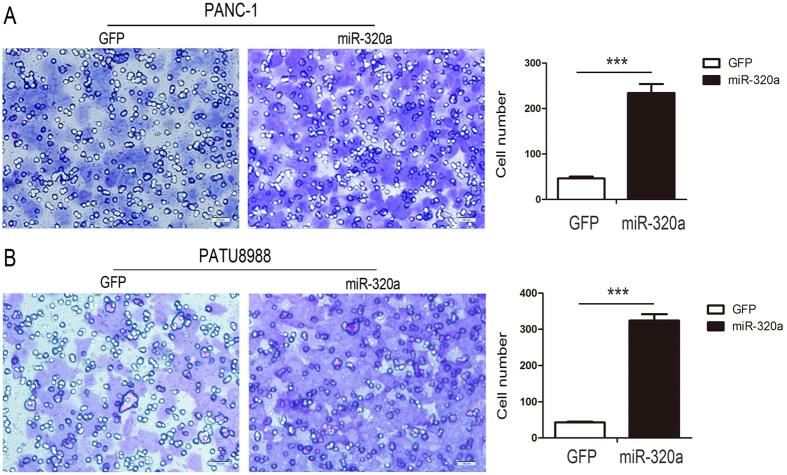
Over-expression of miR-320a promotes pancreatic cancer Cell invasion. (**A**) PANC-1 cells were infected with lenti_miR-320a or lenti_GFP for 24 hours, and transwell invasion assay were performed. The invasive cells per field is shown. (**B**) PATU8988 cells were infected with lenti_miR-320a or lenti_GFP for 24 hours, and transwell invasion assay were performed. The number of invasive cells per field is shown. All data are presented as the means ± SD. ***p < 0.001.

To further confirm the function of miR-320a in pancreatic cancer cells, we investigated the effects of miR-320a on cell invasion, which is important for malignant progression and metastases, using a Matrigel invasion assay. We used PANC-1and PATU8988 cells with transwell assays for these experiments. PANC-1 cells with miR-320a over-expression exhibited significantly increased cell invasion (5.4-fold) compared with the control cells at 24 h (***p < 0.0001, Fig. 4A). PATU8988 cells with miR-320a over-expression also exhibited markedly increased cell invasion (6.6-fold) at 24 h compared with the control cells (***p < 0.0001, Fig. 4B). These findings revealed that miR-320a was critically involved in invasion characteristics in pancreatic cancer cells. Altogether, our results verified that miR-320a played an important role in modulating 5-FU resistance in human pancreatic cancer cells. Over-expression of miR-320a can promote cell proliferation, migration, and invasion of pancreatic cancer cells, also lead to acquisition of EMT characteristic, which all contribute to 5-FU resistance.

### MiR-320a Targets PDCD4 in pancreatic cancer cells

**Figure 5:**
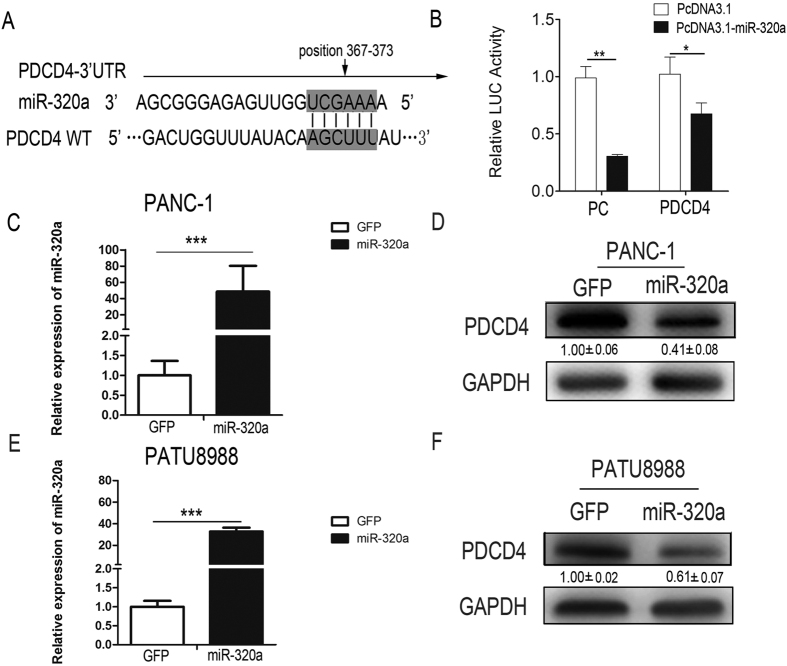
PDCD4 is a direct target of miR-320a in pancreatic cancer cells. (**A**)The prediction of the binding between miR-320a and PDCD4 by Pictar. (**B**) Relative luciferase activity of the indicated PDCD4 reporter constructs in 293T cells. Error bars presented standard deviation obtained from three independent experiments. (**C**) RT-PCR showed the expression of miR-320a in PANC-1 cells after lentivirus infection with lenti_GFP and lenti_miR-320a. (**D**) Western blot analysis showed PDCD4 protein levels in PANC-1 after miR-320a over expression in pancreatic cancer cells. (**E**) RT-PCR results about the expression of miR-320a in PATU8988 cells after lentivirus infection with lenti_GFP and lenti_miR-320a. (**F**) Western bot analysis showed PDCD4 protein levels in PATU8988 after miR-320a over expression in pancreatic cancer cells. All data are presented as the means ± SD. Student’s *t* test (two-tailed) was performed to analyze data from the experiments in triplicate. *P* values < 0.05 were considered significant, as indicated by the asterisks (*p < 0.05, **p < 0.01, ***p < 0.001).

MiRNAs usually exert their functions by negatively regulating the expression of their target genes. The putative targets of miR-320a were predicted using target prediction programs, Target Scan, Pictar and miRanda. Our analysis revealed that PDCD4 was a potential target of miR-320a. The 3′-UTR of PDCD4 messenger RNA contains a complementary site for the seed region of miR-320a (Fig. 5A). To determine whether PDCD4 is a direct target of miR-320a, wild-type 3′-UTR binding sites were cloned into the downstream of firefly luciferase coding region in pMIR-reporter vector. The constructs were then co-transfected with miR-320a over-expression construct or control into 293T cells. The relative luciferase activity was reduced to 60% of the control in miR-320a over-expression group (Fig. 5B). To further determine whether PDCD4 was a direct target of miR-320a in pancreatic cancer cells, we over-expressed miR-320a in the two pancreatic cancer cell lines and detected PDCD4 protein levels. The protein levels of PDCD4 were substantially decreased after ectopic over-expression of miR-320a in pancreatic cancer PANC-1 and PATU8988 cell lines (Fig. 5C,E) as evidenced by western blot assays (Fig. 5D,F). Taken together, these findings indicate that PDCD4 can be negatively regulated by miR-320a, and miR-320a modulates 5-FU resistance in human pancreatic cancer cells by targeting PDCD4.

### PDCD4 could rescue the increased proliferation rate and drug resistance induced by miR-320a in pancreatic cancer cells

**Figure 6:**
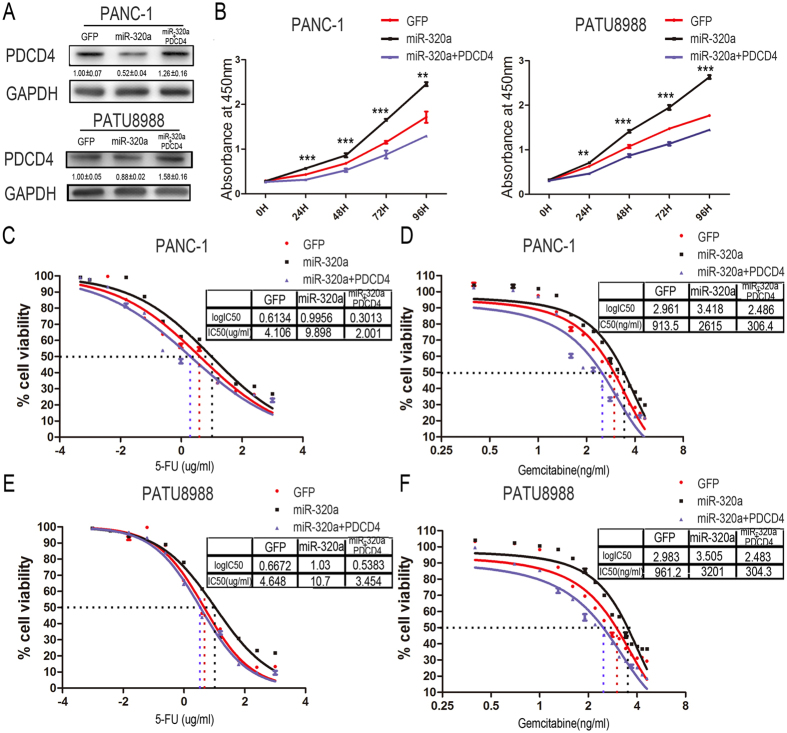
PDCD4 could rescue the increased proliferation rate and drug resistance induced by miR-320a in pancreatic cancer cells. (**A**) Western blot analysis of PDCD4 in pancreatic cancer cells with miR-320a over-expression transfected with PDCD4. (**B**) Proliferation assays by cck-8 at 0, 24, 48, 72 and 96 h after transfected with PDCD4 in PANC-1 and PATU8988 cells. (**C**) Representative curves of growth-inhibitory effects of 5-FU in PANC-1 cells. (**D**) Representative curves of growth-inhibitory effects of gemcitabine in PANC-1 cells. (**E**) Representative curves of growth-inhibitory effects of 5-FU in PATU8988 cells. (**F**) Representative curves of growth-inhibitory effects of gemcitabine in PATU8988 cells. All data are presented as the means ± SD. Student’s *t* test (two-tailed) was performed to analyze data from the experiments in triplicate. *P* values < 0.05 were considered significant, as indicated by the asterisks (**p < 0.01, ***p < 0.001).

To explore whether the promotion of miR-320a on drug-resistance in pancreatic cancer cells was dependent on targeting PDCD4, we performed rescue experiments through over-expressing PDCD4 in PANC-1 and PATU8988 cells infected with lentivirus expressing miR-320a. Western Blot results indicated that transfection of PDCD4 can alleviate the reduction of PDCD4 which induced by miR-320a in pancreatic cancer cells (Fig. 6A). Consistent with the PDCD4 protein level, PDCD4 over-expression rescued the increased proliferation rate by miR-320a in PANC-1 and PATU8988 cells (Fig. 6B). Meanwhile, restoration of PDCD4 expression could attenuate the 5-FU or gemcitabine resistance of pancreatic cancer cells induced by miR-320a. IC50 of 5-FU was evaluated as 4.106 ug/ml for the GFP control, 9.898 ug/ml for miR-320a over-expression group and 2.001 ug/ml for PDCD4 restore group in PANC-1 cells. IC50 of gemcitabine was 913.5 ng/ml for the GFP control, 2615 ng/ml for miR-320a over-expression group and 306.4 ng/ml for PDCD4 restore group in PANC-1 cells. (Fig. 6C,D). The similar results were also observed in PATU8988 cells (Fig. 6E,F). The rescue experiments indicated PDCD4 can rescue the phenotype by miR-320a gain of function and suggested that miR-320a played its function in drug-resistance of pancreatic cancer cells by targeting PDCD4.

## Discussion

As the high mortality and high incidence rate of pancreatic cancer, most studies in pancreatic cancer focus on resectable ones, but few patients could benefit from surgery resection. Therefore chemotherapy become one of the important treatment and auxiliary treatment approaches for patients with pancreatic cancer. Recently, a lot of researches and studies focused on the gemcitabine drug resistance in pancreatic cancer. However, 5-FU which is the earliest pancreatic cancer chemotherapy drug and frequently used in the treatment of pancreatic cancer, is less studied. As we know, 5-FU primary and secondary resistance phenomenon is widespread, which was one of the important factors affected the effect of chemotherapy. In our study, we created 5-FU-resistant pancreatic cancer cell lines and analyzed the expression profile of miRNAs using miRNA microarray to systematically screen the miRNAs involved in the induction of 5-FU drug resistance, and we demonstrated miR-320a played important role in regulating 5-FU resistance in pancreatic cancer firstly.

Previous studies have shown that miR-320a is dysregulated in several cancers and its potential function has also been partly explored in several studies. For example, miR-320a is associated with liver metastasis in colorectal cancer (CRC) and inhibits tumor invasion by targeting neuropilin1^26^. miR-320a/c/d regulate GNAI1 on hepatocellular carcinoma cell migration and invasion post-transcriptionally^27^. Liu *et al*. recently performed miRNA microarray analysis and the result showed that miR-320b was significantly downregulated in NPC relative to non-cancer nasopharyngitis tissues^28^. However, there are no functional evidence of miR-320a in pancreatic cancer. In this study, we found that over-expression of miR-320a can reduce the sensitivity of the pancreatic cancer cells for 5-FU, promote 5-FU resistance. By the way, we found that over-expressing miR-320a can reduce sensitivity of the pancreatic cancer cells for gemcitabine, also promote gemcitabine resistance.

As we known, the EMT process is fundamental to both physiological and pathological development. An aberrantly activated EMT phenotype is a key event in pancreatic cancer development, progression and drug resistance, and may be relevant to therapeutic approaches. Because EMT is controlled by a complex network of transcriptional regulators, cellular signaling pathways and miRNAs. In our study, we firstly show that miR-320a promotes 5-FU resistance through raising EMT transformation in pancreatic cancer cells, exhibiting as down-regulation of epithelial cells markers (β-cadherin and E-cadherin) and up-regulation of mesenchymal cells markers (Fibronectin, N-cadherin, Vimentin, ZEB1 and Snail2). Thus, future study should investigate and survey the elucidation of the molecular biology of EMT in pancreatic cancer, which may provide insights into the mechanisms of tumor recurrence and metastatic progression and alternative therapeutic approaches with important effects on disease outcome.

The expression of miRNAs has been recognized as integral components of many normal biological processes, such as cell proliferation, differentiation, apoptosis, and drug resistance^23^. Recently, miRNA has been recognized to play a critical role in the regulation of cell apoptosis and chemosensitivity^29^,^30^. MiRNAs can complementarily target the mRNAs of target genes, and thereby degrade or inhibit them from translating into proteins. Thus, the altered expression of miRNAs contributes to a variety of human diseases, such as inherited diseases, heart disease and cancer^31^,^32^. MiR-320a was also demonstrated to enhance the sensitivity of human colon cancer cells to chemotherapy *in vitro* by targeting FOXM1^33^. In our study, we find that over-expression of miR-320a not only induces resistance to 5-FU, promotes EMT molecular marker changes, but also promotes proliferation, migration and invasion of pancreatic cancer cells.

The mechanism by which miR-320a exerts its influence on the development of pancreatic cancer was also investigated in this study. Pictar predicted PDCD4 to be a theoretical target gene of miR-320a. We found that miR-320a directly targeted the 3′-UTR of PDCD4 mRNA and repressed its expression. Programmed cell death 4 (PDCD4), a new tumor suppressor, has been documented to be a potential diagnostic tool and treatment target for neoplasia due to the inhabitation of tumor promotion/progression and metastasis. Expressions of PDCD4 were significantly lower in cancers specimens than in noncancerous tissues^34^. In this study, we firstly clarified miR-320a promoted pancreatic cancer cells proliferation and 5-FU resistance in pancreatic cancer cells by targeting PDCD4. Recent study have reported that miR-21 participated in the 5-FU resistance of pancreatic cancer cells by regulating the PDCD4^35^. So we speculated that miR-21 and miR-320a cooperatively contributed to drug-resistance of pancreatic cancer cells through down-regulating PDCD4 together. PDCD4 was also reported to participate in the progress of apoptosis^36^. So we think miR-320a could suppress cell apoptosis by inhibiting PDCD4 and further contribute to drug-resistance, which will be studied in future.

In conclusion, we found that miR-320a was up-regulated in 5-FU resistant pancreatic cancer cells and that miR-320a could promote pancreatic cancer cell proliferation, migration and invasion then contributed to the increased 5-FU resistance. These data suggested that miR-320a might serve as a potential target for pancreatic cancer therapy, and was suitable for establishing some quicker and easier clinical test method. Therefore, the identification of miRNAs which have key roles in the process of drug resistance in pancreatic cancer and clarification of the role of specific molecule is helpful for us to understand pancreatic cancer chemotherapy drug resistance process in the future.

## Materials and Methods

### Cell lines and cultures

The human pancreatic cancer PANC-1 cell line and PATU8988 cell line were obtained from Department of general surgery, Peking Union Medical College (Beijing, China) and 293TN cells were obtained from American Type Culture Collection. PANC-1, 293TN were cultured in Dulbecco’s modified Eagle medium (Invitrogen, USA) supplemented with 10% fetal bovine serum (FBS, from PAA Laboratories, USA), 100 U/ml penicillin, and 100 mg/ml streptomycin. While PATU8988 cells were maintained in RPMI 1640 medium (PAA) supplemented with 10% FBS (PAA). All of the cells were maintained in a 5% CO_2_-humidified atmosphere at 37 °C.

### MiRNA microarray and data analysis

Two groups of miRNA microarrays were carried out with illumina microRNA Expression Beadchip (Human V2) for PATU8988 and PATU8988-5-FU cells. Two biological replicates for each group were performed. MicroRNA gene expression levels were calculated using the PLIER algorithm after a quantile normalization. For differentially expressed miRNAs, we considered a significant change as fold change was >2, or <0.5, P-value was <0.05. MicroRNA microarray data has been deposited in the Gene Expression Omnibus database under accession number GSE30380.

### RNA isolation and quantitative real-time PCR

Total RNA was extracted from human pancreatic cancer PANC-1 cell line and PATU8988 cell line using Trizol reagent (Invitrogen, CA, USA) according to the manufacturer’s instructions. RNA was quantified by absorbance at 260 nm and cDNA was synthesized by M-MLV reverse transcriptase (Invitrogen) from 2 μg of total RNA. Oligo (dT) 18 was used as the RT primers for reverse transcription of mRNA. A stem-loop RT primer was used for the reverse transcription of miRNA. For mRNAs and miRNAs, quantitative real-time PCR was carried out in a Bio-Rad CFX96 real-time PCR System (Bio-Rad, CA, USA) using SYBR Premix Ex Taq kit (Takara, Dalian, China) according to the manufacturer’s instructions. The PCR conditions were as follows: 95 °C for 1 min, followed by 40 cycles of 95 °C for 30 s, 60 °C for 30 s and 72 °C for 1 min. For mRNAs, the data were normalized using the endogenous GAPDH control. For miRNAs, U6 snRNA was used as the endogenous control.

### Construction and transduction of recombinant lentivirus

The pMIRNA1 plasmid and pPACKH1 lenti_vector packaging kit were purchased from SBI (System Biosciences, USA). A 500 bp DNA fragment flanking miR-320a was inserted into the downstream of CMV promoter in pMIRNA1 to generate pMIRNA1-miR-320a. Viral packaging was performed according to the manufacturer’s instructions. Virus particles (lenti-miR320a and lenti_GFP) were harvested and concentrated using PEG-it Virus Precipitation Solution (SBI). Virus titer was determined in 293TN cells using global ultrarapid lentiviral titer kit (SBI). For gene transduction into PATU8988 and PANC-1 cells, the recombinant virus were added to the culture medium of the cells as MOI = 3~5.

### Construction and transfection of expression plasmids

The coding sequence of PDCD4 were obtained and inserted into pcDNA3.1 (+) plasmid. A 500-bp DNA fragment flanking pre-miR-320a was inserted into pcDNA3.1(+) plasmid. The recombinant expression plasmids were transfected into PATU8988 and PANC-1 cells using lipid reagents (Qiagen, China). The transfection efficiency was confirmed by RT-PCR.

### *In vitro* cytotoxicity tests

5-Fluorouracil and Gemcitabine was purchased from Eli Lilly and Company (USA). PATU8988 cells were plated in triplicate at 8 × 10^3^ cells per well in 96-well plates. In the same way, PANC-1 cells were plated in triplicate at 1 × 10^4^ cells per well in 96-well plates. Four hours later, 5-FU (four-fold serial dilution, from 1 × 10^3^ to 9.54 × 10^−4^μg/ml) /gemcitabine(two-fold serial dilution, from 4.096 × 10^4^ to2.5 ng/ml) was added and incubated for 72 hours.

### Cell Proliferation Assay

2.5 × 10^3^ cells of both PATU8988 and PANC-1 cells were split into 96-well plates. The cells were treated with 10 μl CCK-8 (Dojindo, Japan) for 2 hours, followed by a cell proliferation assay. Proliferation rates were determined at 0, 24, 48, 72, 96 hours after seeding. All experiments were performed at least three times.

### Wound Healing Assay

A wound healing assay was performed to examine the capacity of cell migration. After the PATU8988 and PANC-1 cells grew to 90–95% confluence in 24-well plates, a single scratch wound was generated with a 200 μl disposable pipette tip. The scratch wounds were photographed at 0, 12, 24, 36 h hours, with a Nikon inverted microscope with an attached digital camera, and their widths were quantitated with the Image J software. The data were plotted as the percentage of wound opening, setting the initial scratch width as 100%. The results are presented as the mean means ± SD of triplicate measurements per condition in three independent experiments.

### Cell Invasion Assay

The invasive behaviors of the cells were tested using a Matrigel transmembrane invasion assay. Transwell chambers (Millipore) (8 mm pore size) were coated with Matrigel (15 mg/filter). Cells in serum-free medium were plated into the upper chamber and bottom wells were filled with complete medium. Cells were allowed to invade across the Matrigel-coated membrane for 24 h at 37 °C in 5% CO_2_. After 24 hours incubation, cells were removed from the upper surface of the filter by scraping with a cotton swab. The invaded cells that adhered to the bottom of the membrane were fixed with methanol and stained with the 0.1% crystal violet and counted. The number of cells that penetrated the membrane was determined by counting the mean cell number of five randomly selected high power fields.

### Luciferase reporter assay

For miRNA target analysis, the 293TN cells were co-transfected with 0.4 ug of the reporter construct, 0.02 ug of pRL-TK-Renilla luciferase plasmid (Promega, Madison, WI, USA), and miR-320a over-expression construct or control. Cells were harvested 48 h post-transfection and assayed with Dual Luciferase Assay (Promega) according to manufacturer’s instructions. All transfection assays were carried out in triplicates.

### Western blotting

Protein concentration was determined by BCA protein assay kit (Bio-Rad, Italy) and cell lysates were subjected to SDS/PAGE (10% separation gel) and transferred onto a PVDF membrane. The following antibodies were used for Western blot: GAPDH Rabbit mAb antibody (Santa Cruz Biotechnology), PDCD4 Rabbit mAb antibodies (Cell Signal Technology, Danvers, MA, USA). We evaluated the relative expression of protein by Gel-pro Analyzer.

### Statistical analysis

Student’s *t* test (two-tailed) was performed to analyze data from the experiments in three triplicates. All data are presented as the means ± SD. *P* values < 0.05 were considered significant, as indicated by the asterisks (*p < 0.05, **p < 0.01, ***p < 0.001).

## Additional Information

**How to cite this article**: Wang, W. *et al*. MicroRNA-320a promotes 5-FU resistance in human pancreatic cancer cells. *Sci. Rep*. **6**, 27641; doi: 10.1038/srep27641 (2016).
